# Exploring the link between essential tremor and Parkinson’s disease

**DOI:** 10.1038/s41531-023-00577-y

**Published:** 2023-09-15

**Authors:** Sang-Won Yoo, Seunggyun Ha, Chul Hyoung Lyoo, Yuna Kim, Ji-Yeon Yoo, Joong-Seok Kim

**Affiliations:** 1 Department of Neurology, College of Medicine, The Catholic University of Korea, Seoul, Republic of Korea; 2 Division of Nuclear Medicine, Department of Radiology, College of Medicine, The Catholic University of Korea, Seoul, Republic of Korea; 3 Department of Neurology, Gangnam Severance Hospital, Yonsei University College of Medicine, Seoul, Republic of Korea

**Keywords:** Parkinson's disease, Neurodegeneration

## Abstract

Epidemiological studies have reported a link between essential tremor (ET) and Parkinson’s disease (PD). Recent studies have suggested ET as a possible neurodegenerative disease whose subgroup contained Lewy bodies in the brainstem, as in PD. PD with antedated ET (PD_conv_) might exhibit traits different from those of the pure form of ET or PD. This study aimed to unveil the interplay between PD and premorbid ET, which might be the core pathobiology that differentiates PD_conv_ from PD. The study included 51 ET, 32 PD_conv_, and 95 PD patients who underwent positron emission tomography using ^18^F-N-(3-fluoropropyl)-2beta-carbon ethoxy-3beta-(4-iodophenyl) nortropane and ^123^I-meta-iodobenzylguanidine myocardial scintigraphy to analyze central dopaminergic and peripheral noradrenergic integrity. The results show that PD_conv_ group followed the typical striatal pathology of PD but with a delay in noradrenergic impairment as it caught up with the denervating status of PD a few years after PD diagnosis. Whereas the two PD subtypes displayed similar patterns of presynaptic dopamine transporter deficits, ET patients maintained high densities in all subregions except thalamus. Presynaptic dopaminergic availability decreased in a linear or quadratic fashion across the three groups (ET vs. PD_conv_ vs. PD). The age at onset and duration of ET did not differ between pure ET and PD_conv_ patients and did not influence the striatal monoamine status. The myocardium in PD_conv_ patients was initially less denervated than in PD patients, but it degenerated more rapidly. These findings suggest that PD_conv_ could be a distinctive subclass in which the pathobiology of PD interacts with that of ET in the early phase of the disease.

## Introduction

Essential tremor (ET) is a chronic progressive disease that can occur with or without non-motor features such as depression, cognitive deficits, and sleep disorders^1,2^. Accumulating evidence indicates that ET might be a neurodegenerative disease with an emphasis on the cerebellum, leading some to refer to it as ‘*Purkinjopathy*,’ despite some challenges to that view^3–5^.

Epidemiologic and neuropathologic reports also suggest a close relationship between ET and PD, although some disagreements exist^6,7^. Researchers argue that some ET patients develop PD, but not vice versa, indicating unidirectional disease conversion^7,8^. The pathophysiology of ET might predispose some individuals to PD by spreading of Lewy body pathology found in the brainstem^8,9^. Given that various sites of origin can lead to diverse PD phenotypes, the evolution of ET into PD might contribute to the development of tremor-dominant PD.

^123^I-Meta-iodobenzylguanidine (^123^I-MIBG) myocardial scintigraphy has been used to explain the pathobiology of PD by visualizing preclinical incidental Lewy body disease and rostrocaudal temporal gradients of Lewy bodies^10,11^. Denervated myocardium, which reflected central dopaminergic deterioration, was investigated to predict motor complications^12^. Those findings indicate that repeated ^123^I-MIBG myocardial scintigraphy measurements can be used to evaluate the overall pathologic burden of PD and its dynamic changes. Cardiac sympathetic integrity also has been employed to discriminate among neurodegenerative disorders^13^.

Based on the assumption that a subtype of ET could be prone to develop into PD^8^, this study investigated the interplay of ET and PD by assessing dopaminergic and noradrenergic biomarkers. PD patients with antedated ET (PD_conv_) might exhibit traits different from those in the pure forms of ET and PD; analyses of longitudinal changes in myocardial innervation, in particular, might show differences between PD_conv_ and PD. If the pre-existing ET of PD_conv_ shapes the course of cardiac denervation differently from that found in pure PD, the study of PD_conv_ might show how ET influences PD pathobiology.

## Results

### Baseline characteristics

Tables 1 and 2 summarize the baseline characteristics of the study populations. PD patients with antedated ET were the oldest group at the time of diagnosis. The distribution of late-onset ET (LOET) did not differ between the ET and PD_conv_ groups, nor did the preceding duration of ET. Within the LOET group (*n* = 39), the distribution of ET durations (≤5 vs. ≤10 vs. >10) did not differ (Fisher’s exact test; *P*-value 0.104). The proportions of males and females did not differ significantly among the groups. A family history of ET was observed more frequently in both the ET and PD_conv_ groups than in the PD group. The PD_conv_ and PD groups showed a higher association with PD inheritance than the ET group, but that tendency did not reach statistical significance. The median Unified Parkinson’s Disease Rating Scale (UPDRS) Part III score did not differ between PD groups.

**Table 1:** Clinical characteristics.

	ET^a^ (*n* = 51)	PD_conv_^b^ (*n* = 32)	PD^c^ (*n* = 95)	Test statistics (F or t)	*P* value	Effect size	Post-hoc test^d^
Age at diagnosis, years+	70.8 ± 7.2	73.0 ± 8.7	67.4 ± 9.6	5.9	0.003	0.063	b > c**
LOET, *n* (%)	22 (43.1)	17 (53.1)	-	-	0.498	0.097	
Sex, female, *n* (%)	34 (66.7)	18 (56.3)	55 (57.9)	-	0.536	0.086	
Prior ET duration at initial diagnosis, years					0.185	0.205	
3 <, ≤ 5, *n* (%)	22 (43.1)	8 (25.0)	-	-			
5<, ≤ 10, *n* (%)	9 (17.6)	10 (31.3)	-	-			
> 10, *n* (%)	20 (39.2)	14 (43.7)	-	-			
Total follow-up duration, months, median (IQR)	6.0 (29.0)	39.0 (50.8)	71.0 (12.5)	109.6 (*χ*^2^)	<0.001	0.619	a < b**, b < c***, a < c***
Disease duration at baseline, months (IQR)	-	14.0 (19.1)	12.0 (15.5)	1370.0 (*U*)	0.403	0.099	
Disease duration at the last follow-up, months	-	52.7 ± 28.8	86.0 ± 16.7	1.6	<0.001	1.629	
Hypertension, *n* (%)	24 (47.1)	18 (56.3)	41 (43.2)	-	0.453	0.096	
Diabetes mellitus, *n* (%)	12 (23.5)	7 (21.9)	13 (13.7)	-	0.243	0.120	
Dyslipidemia, *n* (%)	20 (39.2)	9 (28.1)	27 (28.4)	-	0.381	0.106	
Non-smoker, *n* (%)	49 (96.1)	32 (100.0)	93 (97.9)	-	0.645	0.089	
Fist degree family history of ET, *n* (%)	9 (17.6)	6 (18.8)	0 (0.0)	-	<0.001	0.308	
First degree family history PD, *n* (%)	1 (2.0)	3 (9.4)	3 (3.2)	-	0.247	0.081	
UPDRS, Part III, median (IQR)	-	18.0 (10.8)	13.0 (10.5)	1185.5 (*U)*	0.063	0.220	
Early H/M_***initial***_ ratio	-	1.65 ± 0.37	1.51 ± 0.27	2.3	0.024	0.466	
Delay H/M_***initial***_ ratio	-	1.60 ± 0.41	1.48 ± 0.33	1.8	0.077	0.365	
Washout rate_***initial***_, %	-	3.11 ± 6.56	2.60 ± 8.98	0.3	0.765	0.061	
Abnormal early H/M ratio, *n* (%)	-	18 (56.3)	74 (77.9)	-	0.023	0.210	
Abnormal delay H/M ratio, *n* (%)	-	21 (65.6)	73 (76.8)	-	0.246	0.111	
^131^I-MIBG scan each interval, months	-	40.5 ± 20.5	28.7 ± 8.3	2.0	0.074	0.753	
Number of ^131^I-MIBG scan, *n* (%)							
1	-	20 (62.5)	26 (27.4)				
2	-	12 (37.5)	48 (50.5)				
≥3	-	-	21 (22.1)				

*LOET* Late-onset essential tremor, *UPDRS* Unified Parkinson’s Disease Rating Scale, *H/M* heart-to-mediastinum, ^*123*^*I-MIBG* ^123^I-meta-iodobenzylguanidine.

Data is shown as mean ± standard deviation unless remarked otherwise.

Independent *t*-test or Welch’s *t*-test, one-way analysis of variance (ANOVA), Mann-Whitney or Kruskal-Wallis tests were performed for continuous variables, and Fisher’s exact test was utilized for categorical variables to discern between-group differences. Test statistics of F or t-distribution are labeled except for the Mann-Whitney (*U*) and Kruskal-Wallis test (*χ*^2^). Eta squared (*η*^2^) and cohen’s *d* for ANOVA and *t*-tests, Cramer’s V for Fisher’s exact test, rank biserial correlation for Mann-Whitney and epsilon squared (*ε*^2^) for Kruskal-Wallis test are shown for effect sizes.

^a^*ET* Essential tremor.

^b^*PD*_*conv*_ Parkinson’s disease converter.

^c^*PD* Parkinson’s disease.

^d^Multiple comparisons were adjusted by Tukey or Dwass-Steel-Critchlow-Fligner methods, as appropriate.

+ The age at diagnosis for PD_conv_ refers to the age when PD was confirmed.

*p < 0.05; **p < 0.01; ***p < 0.001.

**Table 2:** Association between duration of ET and occurrence of disease type, stratified by the age of onset.

		ET duration before diagnosis either ET or PD_conv_			*P*-value	Effect size	*P*-value for trend (*χ*^2^, df)
		≤5	≤10	>10			
LOET	ET	16 (72.7)	4 (18.2)	2 (9.1)	0.104	0.331	0.121 (2.4, 1)
	PD_conv_	7 (41.2)	8 (47.1)	2 (11.8)			
Non-LOET	ET	6 (20.7)	5 (17.2)	18 (62.1)	0.510	0.201	0.187 (1.7, 1)
	PD_conv_	1 (6.7)	2 (13.3)	12 (80.0)			
					Overall *P*-value		
					0.185		0.254 (1.3, 1)

Values are represented as *n* (%).

Fisher’s exact test was utilized to investigate between-group associations and Mantel-Haenszel test was performed to observe any trend across increasing duration of ET by stratum of LOET and disease group. Cramer’s V for Fisher’s exact test is shown for effect sizes.

LOET Late-onset essential tremor, *χ*^2^ chi-squared, df degree of freedom.

The overall heart-to-mediastinum (H/M) uptake ratios were below the reference level for both the early and delayed ratios. The initial H/M uptake ratio was higher in PD_conv_ patients than PD patients, particularly in the early phase (PD_conv_ vs. PD: Early H/M ratio, 1.65 ± 0.37 vs. 1.51 ± 0.27; Delayed H/M ratio, 1.60 ± 0.41 vs. 1.48 ± 0.33; *P*-values, 0.024, 0.077, respectively; Supplementary Fig. 1). A significant portion of PD patients exhibited a high prevalence of abnormal denervation, especially when assessed using the early H/M uptake ratio.

### Presynaptic dopamine transporter density characteristics across disease groups

The results of an analysis of covariance of subregional presynaptic monoamine availability partialized by the age at diagnosis are summarized in Table 3. The standardized uptake value ratios (SUVRs) in both PD_conv_ and PD patients were comparable, whereas ET patients had noticeably higher SUVRs. The SUVRs of the thalamus were similar across all three groups. The differences in SUVRs between ET and PD_conv_ patients were not influenced by LOET status (Supplementary Table 1).

**Table 3:** Subregional standardized uptake value ratio (SUVR) differences across groups.

Subregional SUVR	ET^a^ (*n* = 51)	PD_conv_^b^ (*n* = 32)	PD^c^ (*n* = 95)	Test statistics (F)	*P* value_adj_	Effect size	Post-hoc test^d^
B. Caudate	5.25 ± 0.19	4.37 ± 0.25	4.25 ± 0.14	9.1	<0.001	0.095	a > b*, a > c***
Anterior	5.73 ± 0.22	4.70 ± 0.29	4.58 ± 0.17	9.0	<0.001	0.093	a > b*, a > c***
Posterior	3.98 ± 0.16	3.44 ± 0.21	3.22 ± 0.12	7.2	0.001	0.076	a > c**
R. Caudate	5.46 ± 0.20	4.48 ± 0.26	4.32 ± 0.15	10.7	<0.001	0.109	a > b*, a > c***
Anterior	5.87 ± 0.23	4.70 ± 0.29	4.56 ± 0.17	11.0	<0.001	0.112	a > b**, a > c***
Posterior	4.27 ± 0.18	3.74 ± 0.23	3.45 ± 0.13	6.9	0.002	0.073	a > c**
L. Caudate	5.06 ± 0.20	4.27 ± 0.25	4.18 ± 0.15	6.9	0.002	0.074	a > b*, a > c**
Anterior	5.60 ± 0.23	4.70 ± 0.29	4.59 ± 0.17	6.5	0.002	0.069	a > c**
Posterior	3.75 ± 0.16	3.20 ± 0.20	3.04 ± 0.12	6.6	0.002	0.070	a > c**
B. Putamen	6.84 ± 0.17	4.15 ± 0.22	3.71 ± 0.13	110.5	<0.001	0.559	a > b***, a > c***
Anterior	7.35 ± 0.21	4.39 ± 0.27	3.83 ± 0.16	91.8	<0.001	0.513	a > b***, a > c***
Posterior	6.55 ± 0.19	3.29 ± 0.24	2.77 ± 0.14	139.7	<0.001	0.616	a > b***, a > c***
R. Putamen	6.76 ± 0.18	4.06 ± 0.23	3.60 ± 0.14	98.8	<0.001	0.532	a > b***, a > c***
Anterior	7.28 ± 0.22	4.27 ± 0.28	3.74 ± 0.16	88.3	<0.001	0.504	a > b***, a > c***
Posterior	6.57 ± 0.21	3.29 ± 0.27	2.76 ± 0.15	112.1	<0.001	0.563	a > b***, a > c***
L. Putamen	6.92 ± 0.18	4.25 ± 0.24	3.81 ± 0.14	94.8	<0.001	0.521	a > b***, a > c***
Anterior	7.42 ± 0.23	4.53 ± 0.30	3.93 ± 0.17	73.2	<0.001	0.457	a > b***, a > c***
Posterior	6.57 ± 0.21	3.29 ± 0.27	2.76 ± 0.15	125.8	<0.001	0.591	a > b***, a > c***
B. Globus Pallidus	4.87 ± 0.11	3.55 ± 0.14	3.36 ± 0.08	65.1	<0.001	0.428	a > b***, a > c***
R. Globus Pallidus	5.05 ± 0.13	3.78 ± 0.17	3.42 ± 0.10	51.8	<0.001	0.373	a > b***, a > c***
L. Globus Pallidus	4.71 ± 0.11	3.35 ± 0.14	3.31 ± 0.08	56.0	<0.001	0.392	a > b***, a > c***
B. Ventral Striatum	6.38 ± 0.17	4.98 ± 0.22	4.84 ± 0.13	27.2	<0.001	0.238	a > b***, a > c***
R. Ventral Striatum	6.17 ± 0.18	4.77 ± 0.22	4.58 ± 0.13	27.8	<0.001	0.242	a > b***, a > c***
L. Ventral Striatum	6.60 ± 0.18	5.20 ± 0.23	5.11 ± 0.14	22.9	<0.001	0.208	a > b***, a > c***
B. Ventral Putamen	5.04 ± 0.13	3.69 ± 0.16	3.52 ± 0.09	49.1	<0.001	0.361	a > b***, a > c***
R. Ventral Putamen	4.91 ± 0.14	3.66 ± 0.18	3.45 ± 0.10	38.0	<0.001	0.304	a > b***, a > c***
L. Ventral Putamen	5.16 ± 0.14	3.73 ± 0.18	3.58 ± 0.10	43.4	<0.001	0.333	a > b***, a > c***
B. Thalamus	1.50 ± 0.02	1.46 ± 0.03	1.45 ± 0.02	1.6	0.214	0.018	
R. Thalamus	1.54 ± 0.02	1.52 ± 0.03	1.48 ± 0.02	2.2	0.120	0.025	
L. Thalamus	1.46 ± 0.02	1.41 ± 0.03	1.43 ± 0.02	1.3	0.274	0.012	

*B* both, *R* right, *L* left, *adj* adjusted.

Data is shown as mean ± standard error.

Analysis of covariance, partialized by age at diagnosis, was performed to observe between-group differences. For regional analyses, multiple comparisons across the regions were adjusted by the false discovery rate (FDR) method. Test statistics of F are labeled. Partial eta squared (*η*^2^) estimates are shown for effect sizes.

^a^*ET* Essential tremor.

^b^*PD*_*conv*_ Parkinson’s disease converter.

^c^*PD* Parkinson’s disease.

^d^Post-hoc pairwise comparisons were adjusted by the Scheffe method.

*p < 0.05; **p < 0.01; ***p < 0.001.

**Fig. 1:**
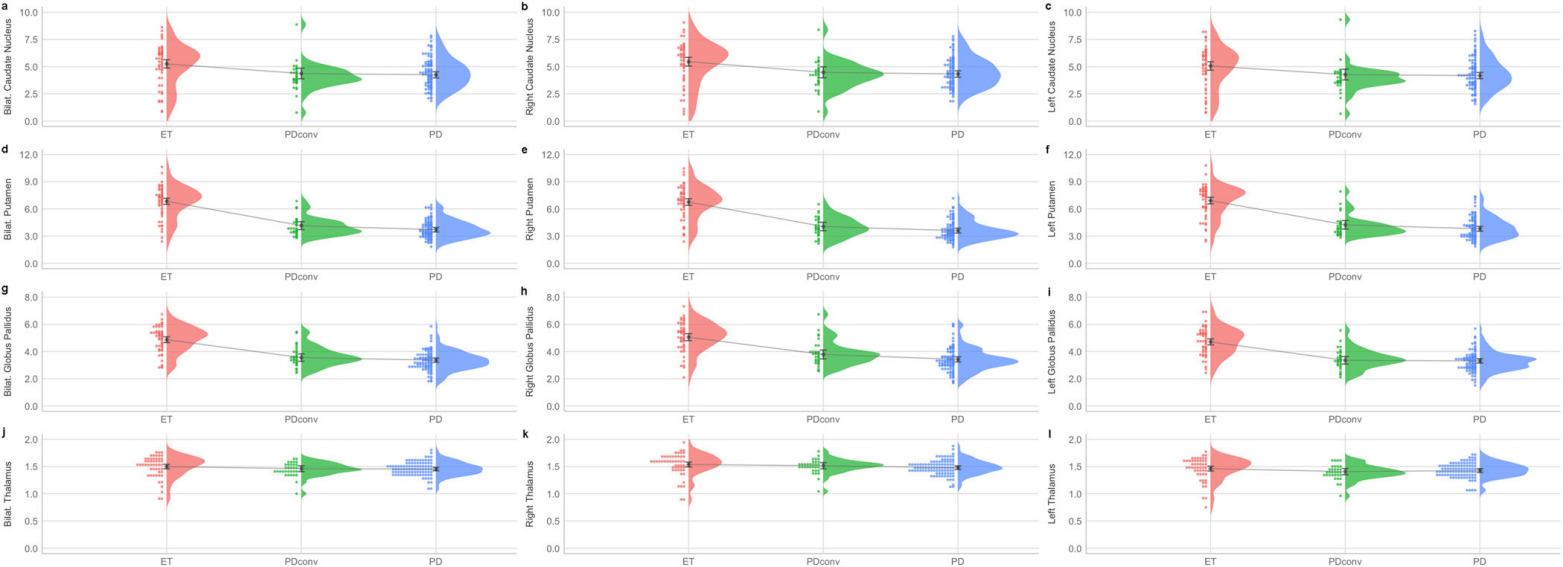
Subregional SUVR trends across groups (ET vs. PD_conv_ vs. PD). **a** Bilateral caudate nucleus, **b** right caudate nucleus, **c** left caudate nucleus, **d** bilateral putamen, **e** right putamen, **f** left putamen, **g** bilateral globus pallidus, **h** right globus pallidus, **i** left globus pallidus, **j** bilateral thalamus, **k** right thalamus, **l** left thalamus. Polynomial contrasts, with age at diagnosis as a covariate, were performed in an analysis of covariance to identify between-group trends. The star represents the estimated means of each group with 95% confidence intervals. SUVR standardized uptake value ratio, Bilat. bilateral, ET essential tremor, PD_conv_ Parkinson’s disease converter, PD Parkinson’s disease.

Polynomial contrasts were calculated to observe any trends across the disease groups. The caudate, putamen, globus pallidus, and thalamus subregions were analyzed (ET vs. PD_conv_ vs. PD; Fig. 1 and Supplementary Table 2). Linear trends were found, signifying that the degree of monoamine density in PD_conv_ patients was between those of ET and PD patients. The putamen and globus pallidus in PD_conv_ patients manifested a sharp decrease of SUVRs compared with ET patients and decelerated decrements when contrasted with PD patients (quadratic trends).

### Longitudinal changes in the ^123^I-MIBG H/M ratio

**Fig. 2:**
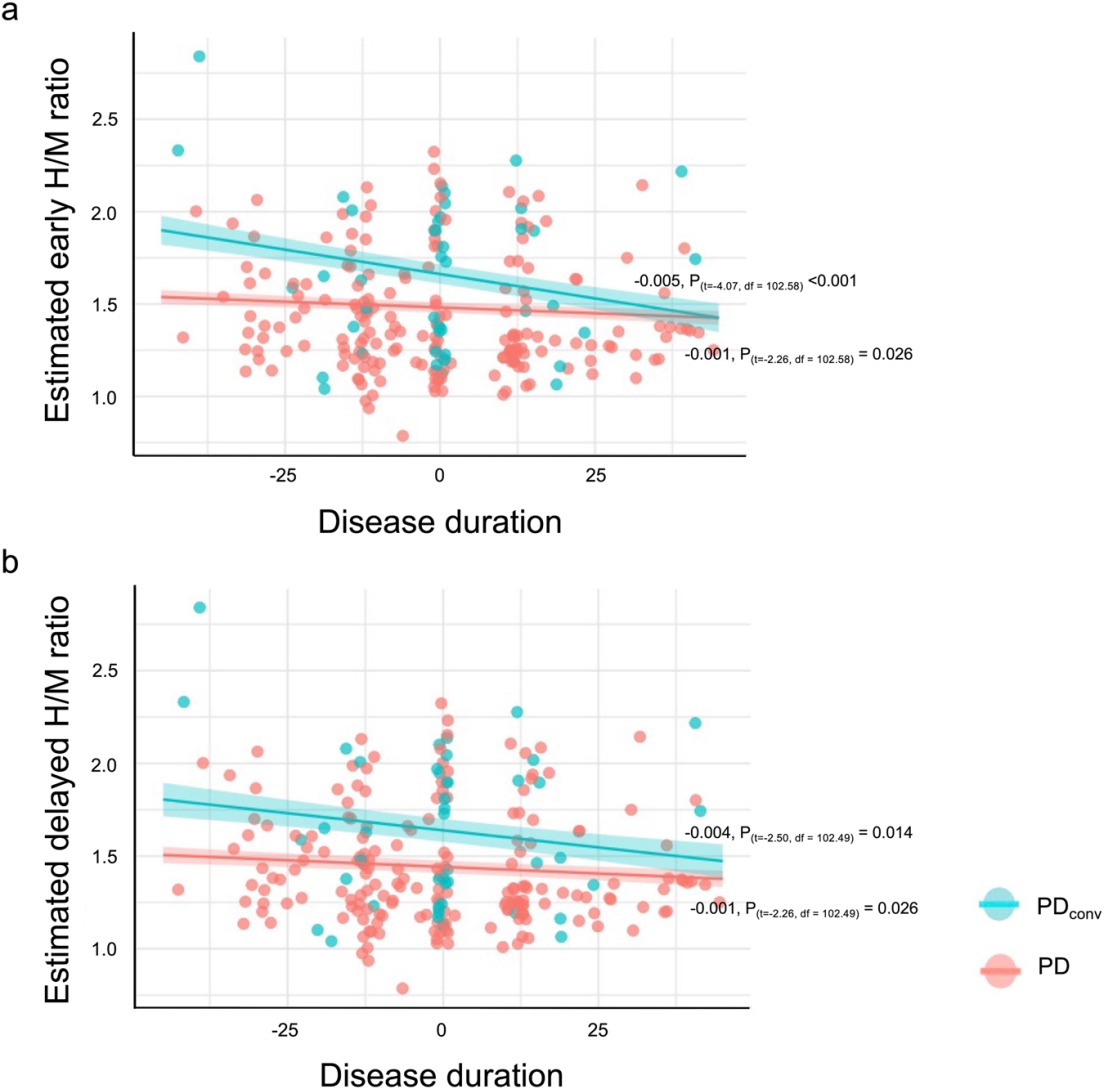
Estimated cardiac denervation stratified by heart-to-mediastinum (H/M) ratio across disease duration. **a** Estimated early H/M ratio, **b** estimated delayed H/M ratio. Shaded areas represent the standard error of the regression lines. The zero on the x-axis signifies the average of the cluster-wise mean-centered disease duration, which is equivalent to 32.3 months. The slope of each regression line was calculated while keeping other independent variables in the model constant. Linear mixed model: Cardiac denervation = Intercept + age at diagnosis + disease duration + disease duration x disease group (reference, PD). Age at diagnosis was grand-mean centered, and disease duration was cluster-wise mean-centered for the analyses. PD Parkinson’s disease, PD_conv_ Parkinson’s disease converter.

A linear mixed model was applied to assess the temporal progression of myocardial scintigraphy. The estimated cardiac denervation trajectory was contrasted between PD_conv_ and PD (Fig. 2).

A repeated measures mixed model of the ^123^I-MIBG H/M ratio revealed a significant inverse linear growth curve against disease duration (Early H/M ratio vs. Delayed H/M ratio; coefficients, −0.003 vs. −0.003; *P*-value, <0.001 vs. 0.002; Supplementary Table 3). The negative association between the H/M ratio and disease duration differed across disease subtypes, particularly in the early H/M ratio group (Early H/M ratio vs. Delayed H/M ratio; coefficients, −0.004 vs. −0.002; *P*-values, 0.005 vs. 0.163; Supplementary Table 3). The difference in early H/M ratios between PD_conv_ and PD neared zero after approximately 77.3 months (*x* = *0* equals 32.3; the difference is estimated to be zero at *x* = *45*, which is equivalent to a disease duration of 77.3 months; Fig. 2). The between-group contrast estimations in the delayed H/M ratio did not converge within the study period, but they did demonstrate a narrowing growth curve. The decay rate of PD was the same across H/M ratios, whereas PD_conv_ had a steeper decline (Fig. 2). H/M ratios showed significant random variance among patients (Early H/M ratio vs. Delayed H/M ratio; Intercept; Intraclass correlation 0.758 vs. 0.776; *χ*^2^_(1)_ = 72.5 vs. 81.3; *P*-value, <0.001; Supplementary Table 3).

## Discussion

In this study, central and peripheral monoamine integrity was compared among ET, PD with preceding ET (ET → PD; PD_conv_), and PD patients. Presynaptic dopaminergic transporter densities did not differ between the two PD subtypes, but ET patients maintained the highest monoamine densities in all subregions except the thalamus. Incremental presynaptic dopaminergic denervation gradients were observed in the striatum, globus pallidus, and thalamus across ET, PD_conv_, and PD patients. The myocardium in patients with pure PD showed more severe initial denervation than in those with PD_conv_, but the rate of denervation was estimated to be more rapid in the PD_conv_ group. These attributes suggest that PD_conv_ could be a distinctive subtype of PD in which the latent influence of ET affects its pathobiology during its progression.

PD patients with preceding ET were significantly older than PD patients, but the age at PD diagnosis was comparable to the age of ET diagnosis. This might reflect the biased nature of the disease entity: individuals with prolonged ET and older onset whose disease converted to PD would be included in the PD_conv_ group. The older onset of PD_conv_ accorded with previous studies^7,14^.

The tremor preceding PD_conv_ might be assessed as merely aging-related tremor^15^, but ET should not be excluded based on age. Pathologic evidence failed to differentiate between ET with younger and older onset, and no suitable age cutoff has been established to separate ET based on age-related symptoms^16,17^. Our data about the age at PD_conv_ onset refer to the age at which PD was confirmed, not ET. About 75% of this group endured more than five years of ET before PD developed, demonstrating comparable onset of ET in PD_conv_ patients to pure ET patients. Therefore, age was not considered to confound the interpretation of data in this study.

Aging and the prolongation of ET were observed to affect clinical progression^1,17–19^. These features were important indicators of neurodegeneration because advanced age and disease duration correlated with increased prevalence, rapid progression, and pathologic changes^4,17,19^. The definition of LOET was carefully designated to imply the underpinning significance of age for both ET and PD. The cutoff age of 65 years was chosen to encompass the increased prevalence of PD, the incidence of PD from ET, and accelerated clinical progression^6,17,19,20^. In a sub-analysis, ET and PD_conv_ did not differ in terms of late-onset age or duration of ET, which were presumed to have no influence on the interpretation of central and peripheral monoamine status. Moreover, disease duration at PD diagnosis did not differ significantly between PD_conv_ and PD patients. This null difference was also not considered to affect the interpretations.

ET patients retained the highest dopamine transporter densities among the three groups in all subregions except the thalamus. This result was expected because a biochemical study measuring striatal tyrosine hydroxylase, a dopaminergic neuron marker, showed no difference in ET patients compared with controls^21^. When comparing PD_conv_ and PD, similar uptake patterns were observed. This finding suggests that some ET patients with Lewy pathology could be triggered into PD pathobiology, resulting in analogous patterns^8,9,22^.

Imaging evidence has suggested that ET could be a neurodegenerative disorder. ET patients were found to have a subtle dopaminergic deficit throughout the whole striatum, and their estimates of dopamine uptake were located below the control level and above the level seen in PD^7,22^. Within the context of the unidirectional evolution of ET into PD_conv_ and post-mortem reports that tremor-dominant PD had milder neurodegeneration^8,9,23^, trend analyses were conducted with PD_conv_ placed between the pure forms of ET and PD. Those analyses revealed a descending gradient of central monoamine density, with accelerated denervation observed in the putamen and globus pallidum. The accelerating presynaptic degeneration of the putamen and globus pallidus as ET progressed to PD was in line with previous studies that showed the greatest deficits in those areas in PD^22,23^. These findings imply that some ET could be a pre-stage of PD that might surpass the threshold for syndromic advancement. By the time it does, nigrostriatal degeneration could reach the sub-level of pure PD, with some characteristic areas such as the putamen and globus pallidus degenerating at levels comparable to those in PD. These results might support the observation that ET could have a neurodegenerative cause as reflected in PD_conv_. Similar findings were observed when the age at diagnosis was squared and included as a covariate in the models to account for the additional effect of age (Supplementary Tables 4 and 5). The results might further strengthen that the observations were independent of age, despite the fact that the effect of age on the brain would be biologically inseparable.

The markers of cardiac denervation not only sub-classify endophenotypes of PD, but also reflect disease burden and deterioration^13,24,25^. In this research, longitudinal measurements of ^123^I-MIBG were used to assess the progression of PD_conv_ and PD. The baseline peripheral noradrenergic integrity was less affected in PD_conv_, but its denervation progressed more rapidly, catching up with the denervated myocardium seen in pure PD after approximately six years. The milder impairment observed in PD_conv_ patients indicated a lesser burden shaped by its development from ET, while its faster deterioration hinted at the transfer of influential pathobiology from ET to PD. A previous study asserted that PD patients suffered abrupt vulnerability five years after diagnosis due to nonlinear deterioration^26^. The progression in PD_conv_ patients of our study appears to lag by one year compared to that of the previous report. The intersection between the two PD subtypes was interpreted to be delayed by the interplay of ET before its influence was gradually weakened as PD biology progressed.

Central dopaminergic (cross-sectional) and peripheral noradrenergic (longitudinal) biomarkers indicate that ET might act as a neurodegenerative disease that evolves into a milder form of PD; however, after the mid-phase of the disease span, PD pathology appeared to dominate the disease biology. This possible transition of dominance underpinned the accelerated degeneration of the myocardium seen in PD_conv_. The disputable transition could also support the potential neurodegenerative nature of some ET^3,4,8,9^. The transformation to PD would not be conceivable if converted ET did not already possess the same degenerative properties as PD.

This study has several strengths. The diagnosis of ET was strengthened by strictly including only individuals with at least three years of tremor. Previous diagnostic criteria had limitations that resulted in heterogeneity in ET classification, but this study applied the most recent criteria, which minimized this issue^2,27^. Additionally, the total disease duration of PD, which is 86 months on average, consolidates its diagnosis by clinically filtering out neurodegenerative PD-mimics. The mean duration of PD_conv_ was shorter than that of PD but was sufficient to exclude atypical PD because the timely development of PD from ET substantiated the diagnosis of PD^8^. These durations adequately safeguard each diagnosis, so disease-specific homogeneity was maintained for these analyses. Unlike epidemiological studies, ET and PD patients with pre-existing ET were also substantiated by presynaptic dopamine transporter imaging, rather than relying solely on clinical diagnosis. The influential roles of older age and disease duration in neurodegeneration were sub-analyzed and interpreted in this study. The null difference between ET and preceding ET of PD_conv_ is critical to the interpretation of monoamine comparisons, which could influence the pathophysiology of both diseases. Finally, this study used both cross-sectional and longitudinal objective biomarkers in its interpretation. The dopamine (intracranial) and noradrenergic (extracranial) markers are crucial surrogates of PD. These neuroimaging techniques could be used in clinical practice to not only differentiate diagnoses but also predict the risk of conversion and progression. However, the cost-effectiveness and clinical utility of those techniques need further substantiation in future studies.

This study also has a few limitations that restrict its generalizability. First, the registration of ET was performed retrospectively. The application of the recent consensus criteria and preserved monoamine density, however, substantiates its diagnosis. A prospective study that includes an examination of the non-motor prodromal features of PD in the ET cohort would further strengthen the argument. Longitudinal ^123^I-MIBG myocardial scintigraphy of pure ET would also provide in-depth insights into the neurodegenerative model if the data could be analyzed with other groups. Moreover, comparisons of associations between central and peripheral denervation in all three groups, not only cross-sectional but also longitudinal, would be of great value in substantiating the argument. Second, the current study did not include disease-specific clinimetrics. To our knowledge, no suitable tools for measuring disease severity are compatible with both ET and PD, to enable direct comparisons. Third, certain subtypes of PD, such as the tremor-dominant phenotype, might differ in their presentations. Contrasting ET with tremor-dominant PD would strengthen this study’s argument, but the sample size was not sufficient for further stratification. Subdividing tremor-dominant PD during enrollment is required in future studies. However, it is noteworthy that postural and action tremors also frequently occur in the non-tremor dominant types of PD. Caution is needed because no validated method is yet available that address this issue in subclassifying motor phenotypes. Fourth, the frequency of family history in ET was lower than in a previous estimation^2^. The retrospective design used in this study could explain that discordance. However, sporadic onset is more prevalent in older ET^17,18^, which might account for the lower prevalence of familial forms because the study population experienced older onset. Moreover, there is a scant epidemiologic inference that explicitly applied the most recent consensus diagnostic criteria, as was done in this study^2,27^. Fifth, this study did not include a control population. If the study had demonstrated differences with the control, it would have bolstered the argument. A future study that enrolls a control population is required. Lastly, the comparisons of presynaptic dopamine transporter density would have been more valuable if the disease duration of each group could be controlled in the model as the disease duration would be an important, indivisible biological factor that affects the integrity and receptor availability.

In conclusion, the Lewy variant subtype of ET might interact with early PD during its development, though its clinical picture might eventually be dominated by PD pathobiology. This PD subtype with preceding ET represents a distinct subclass of PD, and its existence might lend support to the disputable neurodegenerative model of ET.

## Methods

This study was approved by the Institutional Review Board of Seoul St. Mary’s Hospital. All subjects provided written informed consent to participate. Research was conducted in accordance with relevant guidelines and regulations.

### Patients

This study enrolled 127 de novo drug-naïve PD (including PD who converted from ET) and 51 ET patients diagnosed between July 2015 and November 2022. PD was diagnosed based on the UK Parkinson’s Disease Society Brain Bank criteria, and its diagnosis was supported with positron emission tomography (PET) imaging studies using ^18^F-N-(3-fluoropropyl)-2beta-carbon ethoxy-3beta-(4-iodophenyl) nortropane (^18^F-FP-CIT)^28,29^. PD patients exhibited decreased dopamine transporter uptake in the striatum, primarily in the posterior putamen (Supplementary Fig. 2). Among the PD patients enrolled, 32 had pre-existing ET.

Fifty-one ET patients were retrospectively registered, and their diagnoses were confirmed based on both previous criteria and the 2018 Consensus Statement^27,30^. None of the ET patients demonstrated any dopamine transporter uptake deficits in their PET results, which were visually analyzed by a nuclear medicine specialist (S.H.).

Patients were strictly defined as PD-converters (PD_conv_) if pre-existing ET evolved into PD (ET → PD). Co-existence without a clear temporal association between ET and PD was not allowed. All patients endured at least three years of ET without isolated head/voice tremors or mild parkinsonism before they were diagnosed with ET or PD_conv_^27^. Because the exact duration of the tremor could not often be recalled, the preceding period of ET was subclassified into three groups: 3–5 years vs. 6–10 years vs. >10 years. Patients were classified as LOET if ET first emerged after the age of 65 years. That designation was intended to encompass the previously estimated prevalence of both PD and older onset ET^6,18,20,31^. Patients were monitored every 2–6 months, and their diagnosis was confirmed by two neurologists after the last visit (S.-W.Y. and J.-S.K.).

The following baseline characteristics were investigated: age at diagnosis; sex; ET duration before the diagnosis of ET/PD_conv_; follow-up duration; total disease duration; history of hypertension, diabetes mellitus, or dyslipidemia; smoking status; and family history of first-degree relatives with ET or PD. Motor severity was assessed with UPDRS Part III examination.

Patients were excluded if they had any of the following indications: (1) any symptom or sign of atypical and/or secondary parkinsonism during follow-up; (2) magnetic resonance imaging results suggestive of atypical or secondary parkinsonism; (3) history of diabetic or autonomic neuropathy at the initial evaluation; (4) history of symptomatic stroke that could affect general cognition or performance; (5) history of heart failure or documentation of atrial fibrillation on electrocardiography; and (6) current use of medications known to influence the central dopaminergic, noradrenergic, and/or serotonergic systems at the time of diagnosis.

### ^123^I-MIBG myocardial scintigraphy

^123^I-MIBG scintigraphy was performed using a dual-head camera equipped with a low-energy, high-resolution collimator (Siemens), and data were collected 30 min (early) and 120 min (delayed) after injection of 111 MBq of ^123^I-MIBG. A static image was obtained with a 128 × 128 matrix. Regions of interest were manually drawn around the heart and mediastinum. Tracer uptake was measured within each region of interest to calculate the H/M ratio at the early and delayed time points. The reference values for abnormal early and delayed H/M ratios were defined as <1.70 and <1.78, respectively^32^. The washout rate was calculated as follows: [(early H/M ratio – late H/M ratio)/early H/M ratio] x 100^32^.

All patients were initially evaluated at the time of diagnosis, and they were re-assessed 2–3 times. The interval between clinical and cardiac assessments was 0.9 months (interquartile range, IQR 1.3).

### Imaging acquisition and processing of ^18^F-FP-CIT PET

^18^F-FP-CIT PET images were assessed once at the initial diagnostic evaluation. Brain computed tomography (CT) and ^18^F-FP-CIT PET images were acquired using a Discovery STE PET/CT scanner (Discovery PET/CT 710, General Electric Healthcare, Waukesha, WI, USA). Three hours after an intravenous injection of 3.7MBq/kg of ^18^F-FP-CIT, brain CT scans were obtained for attenuation correction, followed by a 10-minute ^18^F-FP-CIT PET scan. PET images were reconstructed using a fully 3D-ordered subset-expectation maximization algorithm (VUE Point HD) with 4 iterations and 16 subsets. This reconstruction was combined with a Gaussian filter featuring a 3.3-mm full width at half maximum. The images were processed using a 256 × 256 matrix size, a pixel spacing of 0.976 cm * 0.976 cm, and a slice thickness of 3.27 mm.

Statistical Parametric Mapping 8 software (SPM8; Wellcome Trust Centre for Neuroimaging, London, UK) and an in-house automated pipeline program implemented in MATLAB 2015a (MathWorks, Natick, MA, USA) were used for the processing of ^18^F-FP-CIT PET images. For the quantification of striatal uptake, a skull-stripped CT (ssCT) guided image processing method validated in the previous paper was utilized^33,34^. In brief, individual CT images were first skull-stripped by using the SPM segmentation tool with the probabilistic templates for the skull, brain, and cerebrospinal fluid (CSF). ^18^F-FP-CIT PET images were co-registered to brain CT images and then spatially normalized to MNI space with 1 mm isovoxel, with the parameter normalizing ssCT images to the ssCT template^33,34^. By overlaying the in-house striatal volume of interest (VOI) templates derived from the FreeSurfer 5.1 (Massachusetts General Hospital, Harvard Medical School; http://surfer.nmr.mgh.harvard.edu) on each spatially normalized PET image, the striatal subregional and cerebellar uptake binding values were measured. Finally, the regional standardized uptake values ratios (SUVRs) for each side of the caudate, putamen, globus pallidus, and ventral striatum were calculated with the cerebellar cortex as a reference. All the image processing was executed by the previously validated method in the previous works, which provided the relevant templates^33,34^.

The interval between clinical assessment and PET was 0.2 months (IQR 0.8). The interval between ^123^I-MIBG scintigraphy and ^18^F-FP-CIT PET was 0.0 months (IQR 0.9).

### Statistical analyses

Statistical analyses were performed with jamovi software (version 2.3.22; retrieved from https://www.jamovi.org) for Mac. The software is a graphical user interface for R and includes a GAMLj module (version 2.6.6; https://gamlj.github.io) that allows linear mixed modeling.

Descriptive statistics and independent *t*-test, Welch’s *t*-test, analysis of variance (ANOVA), or Kruskal-Wallis test were performed for continuous variables. Categorical variables were examined using Fisher’s exact test. The Mantel-Haenszel test was used to detect any trends between nominal and ordinal scales.

Analysis of covariance (ANCOVA) was applied to assess the between-group differences in subregional SUVRs with age at diagnosis as a covariate. Polynomial contrasts were used to investigate trends across the disease groups: ET vs. PD_conv_ vs. PD. A linear mixed model by restricted maximum likelihood estimation was performed to investigate the pattern of cardiac denervation progression. The intercept was set as a random effect to reflect individual variance. Multiple comparisons were adjusted using the Tukey and Dwass-Steel-Critchlow-Fligner methods for ANOVA and Kruskal-Wallis testing, respectively. In region-based analyses, the false discovery rate method was applied to the multiple ANCOVA tests. The Scheffe method was used when adjusting for post hoc pairwise comparisons among the disease groups. Statistical significance was defined as a two-tailed *p*-value < 0.05.

### Reporting summary

Further information on research design is available in the Nature Research Reporting Summary linked to this article.

### Supplementary information

Supplementary information

Reporting Summary

## Data Availability

Anonymized data generated during the current study are available from the corresponding author on request from individuals affiliated with research or healthcare institutions.
