# Supplementary material for: Exploring the link between essential tremor and Parkinson’s disease
Source: NPJ Parkinsons Dis. 2023 Sep 15;9:134. doi: 10.1038/s41531-023-00577-y (PMC10504235; doi:10.1038/s41531-023-00577-y)
Supplement: Supplementary file 1 — Supplementary information [file 41531_2023_577_MOESM1_ESM.pdf]

Supplementary Figure 1. Between-group differences in Heart-to-Mediastinum (H/M) ratios at the initial assessment

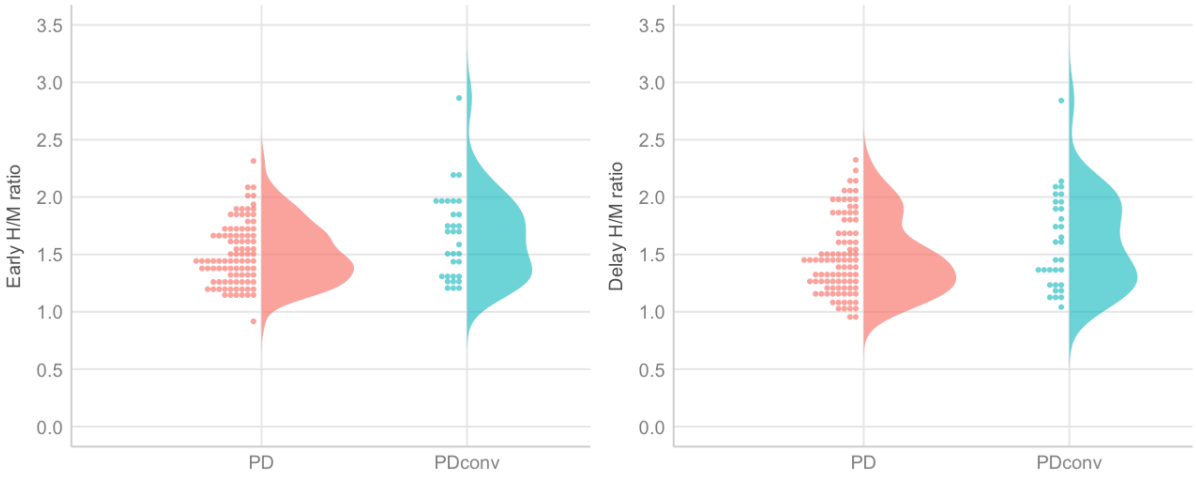

Supplementary Figure 2. Exemplary images of ^{18}F-FP-CIT PET CT of each group at its initial diagnostic workup.

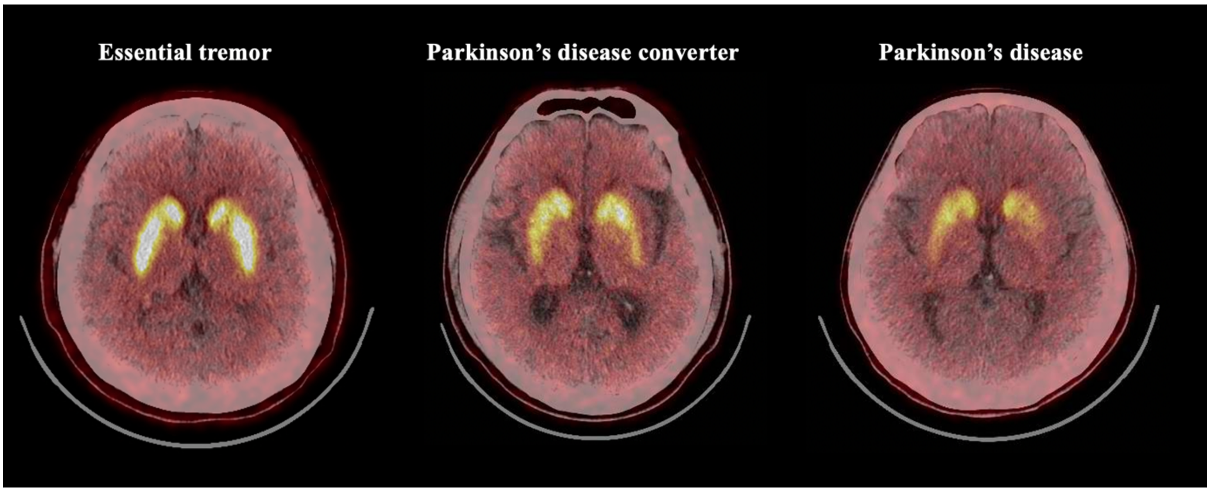

Supplementary table 1. Estimates of subregional standardized uptake value ratio (SUVR) of disease groups stratified by LOET.

		Estimate	SE	β	t	P-value
B. Caudate	Disease groups x LOET	0.194	0.707	0.110	0.274	0.785
R. Caudate	Disease groups x LOET	-0.139	0.738	-0.076	-0.188	0.852
L. Caudate	Disease groups x LOET	0.508	0.708	0.289	0.718	0.475
B. Putamen	Disease groups x LOET	0.742	0.660	0.368	1.124	0.264
R. Putamen	Disease groups x LOET	0.453	0.703	0.220	0.645	0.521
L. Putamen	Disease groups x LOET	1.013	0.664	0.494	1.526	0.131
B. Globus Pallidus	Disease groups x LOET	0.085	0.384	0.08	0.221	0.826
R. Globus Pallidus	Disease groups x LOET	0.222	0.463	0.189	0.480	0.633
L. Globus Pallidus	Disease groups x LOET	-0.035	0.391	-0.032	-0.09	0.928
B. Vent. Striatum	Disease groups x LOET	0.110	0.568	0.076	0.194	0.847
R. Vent. Striatum	Disease groups x LOET	0.107	0.593	0.072	0.181	0.857
L. Vent. Striatum	Disease groups x LOET	0.113	0.571	0.078	0.198	0.843
B. Vent. Putamen	Disease groups x LOET	0.301	0.447	0.249	0.674	0.502
R. Vent. Putamen	Disease groups x LOET	0.189	0.472	0.152	0.401	0.690
L. Vent. Putamen	Disease groups x LOET	0.404	0.499	0.309	0.811	0.420
B. Thalamus	Disease groups x LOET	0.120	0.078	0.673	1.530	0.130
R. Thalamus	Disease groups x LOET	0.128	0.082	0.688	1.566	0.121
L. Thalamus	Disease groups x LOET	0.111	0.085	0.582	1.313	0.193

Linear regression model, controlled of age at diagnosis, was performed to observe the interactions between LOET and disease groups (ET *vs.* PD_{conv}).
Model: SUVRs = Intercept + Age at diagnosis + Disease groups (ET vs. PD_{conv}) + LOET + Disease groups x LOET
Data is shown as mean ± standard error.

SUVR standardized uptake value ratio, B both, R right, L left, Vent ventral, LOET late-onset essential tremor

Supplementary table 2. Subregional standardized uptake value ratio (SUVR) trend across groups (ET *vs.* PD_{conv} *vs.* PD)

SUVR	Contrast	Estimate	SE	t	$P\text{-value}_{adj}$
B. Caudate	linear	-0.711	0.171	-4.162	<0.001
	quadratic	0.311	0.224	1.388	0.250
R. Caudate	linear	-0.800	0.178	-4.505	<0.001
	quadratic	0.338	0.233	1.452	0.250
L. Caudate	linear	-0.626	0.173	-3.608	0.001
	quadratic	0.285	0.227	1.254	0.282
B. Putamen	linear	-2.215	0.152	-14.529	<0.001
	quadratic	0.914	0.200	4.576	<0.001
R. Putamen	linear	-2.233	0.162	-13.747	<0.001
	quadratic	0.917	0.213	4.308	<0.001
L. Putamen	linear	-2.198	0.163	-13.455	<0.001
	quadratic	0.911	0.214	4.257	<0.001
B. Globus Pallidus	linear	-1.064	0.096	-11.122	<0.001
	quadratic	0.459	0.125	3.662	0.001
R. Globus Pallidus	linear	-1.152	0.114	-10.070	<0.001
	quadratic	0.371	0.150	2.476	0.029
L. Globus Pallidus	linear	-0.988	0.098	-10.098	<0.001
	quadratic	0.536	0.128	4.183	<0.001
B. Thalamus	linear	-0.033	0.019	-1.749	0.089
	quadratic	0.013	0.025	0.520	0.659
R. Thalamus	linear	-0.041	0.020	-2.066	0.048
	quadratic	-0.005	0.026	-0.195	0.845
L. Thalamus	linear	-0.025	0.020	-1.232	0.219
	quadratic	0.030	0.026	1.163	0.296

Polynomial contrasts, with covariate of age at diagnosis, was performed in analysis of covariance to discern between-group trends.
Data is shown as mean ± standard error. For regional analyses, multiple comparisons across subregions were adjusted by the false discovery rate (FDR) method.

SUVR standardized uptake value ratio, B both, R right, L left, Vent ventral, SE standard error, adj adjusted

ET, Essential tremor
PDconv, Parkinson's disease converter
PD, Parkinson's disease

*p<0.05; **p<0.01; ***p<0.001

Supplementary table 3. Summary of linear mixed models

Random intercept mixed model with the covariate of age at diagnosis, stratified by the disease (reference, PD)
Model: Cardiac denervation = Intercept + Age at diagnosis + disease duration + disease duration x disease groups
Age at diagnosis was grand mean-centered, and disease duration was clusterwise mean-centered to avoid multicollinearity.

1. Dependent variable: Early H/M ratio
A. Fixed Effects

	Estimate	SE	df	t	P-value
Intercept	1.572	0.029	125.281	53.580	<0.001
Age at diagnosis	0.000	0.003	116.077	0.051	0.959
Disease groups					
PD_{conv} – PD	0.181	0.059	125.932	3.045	0.003
Disease Duration	-0.003	0.001	97.587	-4.631	<0.001
Disease Duration x Disease groups	-0.004	0.001	97.587	-2.846	0.005

Satterthwaite method for degrees of freedom

B. Random Effects

		SD	Variance	ICC
Individual patient	Intercept	0.253	0.064	0.758
Residual		0.143	0.020	

Likelihood Ratio Test	$\chi 2$	df	P-value
Intercept random across individual	72.462	1	<0.001

PD Parkinson's disease, PD_{conv} Parkinson's disease converter, SD standard deviation, ICC Intraclass Correlation, SE standard error, df degree of freedom

2. Dependent variable: Delay H/M ratio
A. Fixed Effects

	Estimate	SE	df	t	P-value
Intercept	1.540	0.035	125.790	44.208	<0.001
Age at diagnosis	-0.001	0.003	117.365	-0.353	0.725
Disease groups					
PD_{conv} – PD	0.197	0.071	126.379	2.796	0.006
Disease Duration	-0.003	0.001	98.483	-3.186	0.002
Disease Duration x Disease groups	-0.002	0.002	98.483	-1.404	0.163

Satterthwaite method for degrees of freedom

B. Random Effects

		SD	Variance	ICC
Individual patient	Intercept	0.303	0.092	0.776
Residual		0.163	0.026	

Likelihood Ratio Test	$\chi 2$	df	P-value
Intercept random across individual	81.337	1	<0.001

PD Parkinson's disease, PD_{conv} Parkinson's disease converter, SD standard deviation, ICC Intraclass Correlation, SE standard error, df degree of freedom

Supplementary table 4. Subregional standardized uptake value ratio (SUVR) differences across groups

Subregional SUVR	ET[a] (n= 51)	PD_{conv}[b] (n= 32)	PD[c] (n= 95)	Test statistics (F)	P $value_{adj}$	Effect size	Post hoc analysis[d]
B. Caudate	5.15 ± 0.19	4.10 ± 0.24	4.40 ± 0.14	7.1	0.002	0.076	a>b**, a>c**
Anterior	5.61 ± 0.22	4.39 ± 0.28	4.75 ± 0.16	7.1	0.002	0.075	a>b**, a>c**
Posterior	3.90 ± 0.16	3.23 ± 0.20	3.34 ± 0.12	5.0	0.010	0.054	a>b*, a>c*
R. Caudate	5.36 ± 0.20	4.24 ± 0.25	4.45 ± 0.15	8.5	<0.001	0.090	a>b**, a>c**
Anterior	5.77 ± 0.23	4.43 ± 0.29	4.71 ± 0.17	9.0	<0.001	0.094	a>b**, a>c**
Posterior	4.20 ± 0.18	3.54 ± 0.22	3.56 ± 0.13	4.8	0.011	0.052	a>c*
L. Caudate	4.94 ± 0.20	3.97 ± 0.25	4.34 ± 0.14	5.4	0.007	0.058	a>b**, a>c*
Anterior	5.46 ± 0.23	4.34 ± 0.29	4.79 ± 0.17	5.0	0.009	0.055	a>b*
Posterior	3.66 ± 0.16	2.98 ± 0.20	3.16 ± 0.12	4.7	0.012	0.051	a>b*, a>c*
B. Putamen	6.80 ± 0.17	4.03 ± 0.22	3.77 ± 0.13	106.4	<0.001	0.551	a>b***, a>c***
Anterior	7.28 ± 0.21	4.22 ± 0.27	3.92 ± 0.16	86.7	<0.001	0.501	a>b***, a>c***
Posterior	6.50 ± 0.18	3.16 ± 0.23	2.84 ± 0.13	136.9	<0.001	0.613	a>b***, a>c***
R. Putamen	6.73 ± 0.18	3.99 ± 0.23	3.64 ± 0.13	97.1	<0.001	0.529	a>b***, a>c***
Anterior	7.23 ± 0.22	4.15 ± 0.27	3.80 ± 0.16	85.2	<0.001	0.496	a>b***, a>c***
Posterior	6.54 ± 0.21	3.20 ± 0.26	2.81 ± 0.15	111.9	<0.001	0.564	a>b***, a>c***
L. Putamen	6.85 ± 0.18	4.09 ± 0.23	3.89 ± 0.14	89.5	<0.001	0.509	a>b***, a>c***
Anterior	7.33 ± 0.23	4.30 ± 0.30	4.06 ± 0.17	67.6	<0.001	0.439	a>b***, a>c***
Posterior	6.47 ± 0.19	3.13 ± 0.24	2.87 ± 0.14	120.9	<0.001	0.583	a>b***, a>c***
B. Globus Pallidus	4.90 ± 0.11	3.63 ± 0.14	3.32 ± 0.08	71.5	<0.001	0.453	a>b***, a>c***
R. Globus Pallidus	5.08 ± 0.13	3.84 ± 0.16	3.39 ± 0.09	55.7	<0.001	0.392	a>b***, a>c***
L. Globus Pallidus	4.74 ± 0.11	3.44 ± 0.14	3.26 ± 0.08	62.7	<0.001	0.420	a>b***, a>c***
B. Ventral Striatum	6.35 ± 0.17	4.89 ± 0.22	4.89 ± 0.13	25.5	<0.001	0.228	a>b***, a>c***
R. Ventral Striatum	6.15 ± 0.17	4.72 ± 0.22	4.61 ± 0.13	26.9	<0.001	0.237	a>b***, a>c***
L. Ventral Striatum	6.55 ± 0.18	5.07 ± 0.23	5.18 ± 0.13	21.0	<0.001	0.196	a>b***, a>c***
B. Ventral Putamen	5.01 ± 0.13	3.63 ± 0.16	3.55 ± 0.09	47.0	<0.001	0.352	a>b***, a>c***
R. Ventral Putamen	4.89 ± 0.14	3.60 ± 0.17	3.48 ± 0.10	36.6	<0.001	0.297	a>b***, a>c***
L. Ventral Putamen	5.13 ± 0.14	3.66 ± 0.18	3.61 ± 0.10	41.4	<0.001	0.323	a>b***, a>c***
B. Thalamus	1.50 ± 0.02	1.45 ± 0.03	1.46 ± 0.02	1.2	0.306	0.014	
R. Thalamus	1.54 ± 0.02	1.51 ± 0.03	1.49 ± 0.02	1.6	0.214	0.018	
L. Thalamus	1.46 ± 0.02	1.41 ± 0.03	1.43 ± 0.02	1.1	0.326	0.013	

Data is shown as mean ± standard error.
Analysis of covariance, partialized by age at diagnosis and its square, was performed to observe between-group differences. Age and its squared values were clusterwise mean-centered to avoid multicollinearity. For regional analyses, multiple comparisons across the subregions were adjusted by the false discovery rate (FDR) method. Test statistics of F are labeled. Partial eta squared ($\eta 2$) estimates are shown for effect sizes.

[a] ET, Essential tremor
[b] PD_{conv}, Parkinson's disease converter
[c] PD, Parkinson's disease
[d] Post hoc pairwise comparisons were adjusted by the Scheffe method.

B both, R right, L left, adj adjusted

*p<0.05; **p<0.01; ***p<0.001

Supplementary table 5. Subregional standardized uptake value ratio (SUVR) trend across groups (ET *vs.* PD_{conv} *vs.* PD)

SUVR	Contrast	Estimate	SE	t	$P\text{-value}_{adj}$
B. Caudate	linear	-0.530	0.169	-3.141	0.004
	quadratic	0.550	0.221	2.488	0.022
R. Caudate	linear	-0.642	0.176	-3.660	0.001
	quadratic	0.547	0.230	2.378	0.026
L. Caudate	linear	-0.423	0.171	-2.471	0.022
	quadratic	0.553	0.225	2.462	0.022
B. Putamen	linear	-2.139	0.151	-14.209	<0.001
	quadratic	1.015	0.197	5.144	<0.001
R. Putamen	linear	-2.187	0.160	-13.640	<0.001
	quadratic	0.978	0.210	4.655	<0.001
L. Putamen	linear	-2.094	0.161	-12.974	<0.001
	quadratic	1.049	0.211	4.961	<0.001
B. Globus Pallidus	linear	-1.113	0.094	-11.844	<0.001
	quadratic	0.395	0.123	3.213	0.003
R. Globus Pallidus	linear	-1.189	0.113	-10.526	<0.001
	quadratic	0.322	0.148	2.176	0.041
L. Globus Pallidus	linear	-1.046	0.095	-10.967	<0.001
	quadratic	0.459	0.125	3.678	0.001
B. Thalamus	linear	-0.025	0.019	-1.371	0.197
	quadratic	0.023	0.024	0.935	0.383
R. Thalamus	linear	-0.035	0.020	-1.798	0.093
	quadratic	0.003	0.026	0.103	0.918
L. Thalamus	linear	-0.016	0.020	-0.790	0.450
	quadratic	0.042	0.026	1.642	0.123

Polynomial contrasts, with covariate of age at diagnosis and its square, were performed in the analysis of covariance to discern between-group trends. Age and its squared values were clusterwise mean-centered to avoid multicollinearity. Data is shown as mean ± standard error. Multiple comparisons across subregions were adjusted by the false discovery rate (FDR) method.

SUVR standardized uptake value ratio, B both, R right, L left, Vent ventral, SE standard error, adj adjusted

ET, Essential tremor
PDconv, Parkinson’s disease converter
PD, Parkinson’s disease

*p<0.05; **p<0.01; ***p<0.001
