# Supplementary material for: Exploring the link between essential tremor and Parkinson’s disease
Source: NPJ Parkinsons Dis. 2023 Sep 15;9:134. doi: 10.1038/s41531-023-00577-y (PMC10504235; doi:10.1038/s41531-023-00577-y)
Supplement: Supplementary file 2 — Reporting Summary [file 41531_2023_577_MOESM2_ESM.pdf]

nature portfolio

Corresponding author(s): Joong-Seok Kim

Last updated by author(s): Sep 1, 2023

Reporting Summary

Nature Portfolio wishes to improve the reproducibility of the work that we publish. This form provides structure for consistency and transparency in reporting. For further information on Nature Portfolio policies, see our Editorial Policies and the Editorial Policy Checklist.

Statistics

For all statistical analyses, confirm that the following items are present in the figure legend, table legend, main text, or Methods section.

n/a	Confirmed	
☐	☒	The exact sample size (*n*) for each experimental group/condition, given as a discrete number and unit of measurement
☐	☒	A statement on whether measurements were taken from distinct samples or whether the same sample was measured repeatedly
☐	☒	The statistical test(s) used AND whether they are one- or two-sided<br>*Only common tests should be described solely by name; describe more complex techniques in the Methods section.*
☐	☒	A description of all covariates tested
☐	☒	A description of any assumptions or corrections, such as tests of normality and adjustment for multiple comparisons
☐	☒	A full description of the statistical parameters including central tendency (e.g. means) or other basic estimates (e.g. regression coefficient) AND variation (e.g. standard deviation) or associated estimates of uncertainty (e.g. confidence intervals)
☐	☒	For null hypothesis testing, the test statistic (e.g. F, t, r) with confidence intervals, effect sizes, degrees of freedom and P value noted<br>*Give P values as exact values whenever suitable.*
☒	☐	For Bayesian analysis, information on the choice of priors and Markov chain Monte Carlo settings
☒	☐	For hierarchical and complex designs, identification of the appropriate level for tests and full reporting of outcomes
☐	☒	Estimates of effect sizes (e.g. Cohen's *d*, Pearson's *r*), indicating how they were calculated

Our web collection on statistics for biologists contains articles on many of the points above.

Software and code

Policy information about availability of computer code

Data collection	Data collection was done with Microsoft Excel. The files were then converted into csv files for statistical programs
Data analysis	For data analysis, jamovi software (version 2.3.22) with the GAMLj module (version 2.6.6).

For manuscripts utilizing custom algorithms or software that are central to the research but not yet described in published literature, software must be made available to editors and reviewers. We strongly encourage code deposition in a community repository (e.g. GitHub). See the Nature Portfolio guidelines for submitting code & software for further information.

Data

Policy information about availability of data

All manuscripts must include a data availability statement. This statement should provide the following information, where applicable:

- Accession codes, unique identifiers, or web links for publicly available datasets
- A description of any restrictions on data availability
- For clinical datasets or third party data, please ensure that the statement adheres to our policy

Anonymized data generated during this study are available from the corresponding author on request from individuals affiliated with research or health care institutions.

Research involving human participants, their data, or biological material

Policy information about studies with human participants or human data. See also policy information about sex, gender (identity/presentation), and sexual orientation and race, ethnicity and racism.

Reporting on sex and gender	Biological attribute was considered in this study; hence, the term sex was used.
Reporting on race, ethnicity, or other socially relevant groupings	The sample is representative of PD population of a single center. It is from our cohort registry that collects data retrospectively and prospectively. Participants with analyzable data were selected from this registry. Thus this population included with patients with Korean nationality.
Population characteristics	The mean age of essential tremor, Parkinson's disease converter and Parkinson's disease was 70.8 ± 7.2, 73.0 ± 8.7, 67.4 ± 9.6 years, and 34 (66.7%), 18 (56.3%), 55 (57.9%) were female, respectively. Disease duration at diagnosis was 14.0 (IQR, 19.1) and 12.0 (IQR 15.5) months for PDconv and PD. Total follow-up duration was 6.0 (IQR, 29.0), 39.0 (IQR 50.8), 71.0 (12.5) for their respective groups of ET, PDconv and PD. The sample is representative of ET and PD population of a single center. It is from our cohort registry that collects data retrospectively and prospectively. Participants with analyzable data were selected from this registry.
Recruitment	ET and PD patients who visited our movement disorder clinics of Seoul St. Mary's Hospital were recruited. As this study enrolled at a single center, this may cause selection bias.
Ethics oversight	This study was approved by the Institutional Review Board of Seoul St. Mary's Hospital.

Note that full information on the approval of the study protocol must also be provided in the manuscript.

Field-specific reporting

Please select the one below that is the best fit for your research. If you are not sure, read the appropriate sections before making your selection.

☐ Life sciences ☒ Behavioural & social sciences ☐ Ecological, evolutionary & environmental sciences

For a reference copy of the document with all sections, see nature.com/documents/nr-reporting-summary-flat.pdf

Behavioural & social sciences study design

All studies must disclose on these points even when the disclosure is negative.

Study description	This study is a cross-sectional and longitudinal observational study with mixed-methods. It contains mainly of quantitative data but also qualitative data.
Research sample	The mean age of essential tremor, Parkinson's disease converter and Parkinson's disease was 70.8 ± 7.2, 73.0 ± 8.7, 67.4 ± 9.6 years, and 34 (66.7%), 18 (56.3%), 55 (57.9%) were female, respectively. Disease duration at diagnosis was 14.0 (IQR, 19.1) and 12.0 (IQR 15.5) months for PDconv and PD. Total follow-up duration was 6.0 (IQR, 29.0), 39.0 (IQR 50.8), 71.0 (12.5) for their respective groups of ET, PDconv and PD. The sample is representative of ET and PD population of a single center. It is from our cohort registry that collects data retrospectively and prospectively. Participants with analyzable data were selected from this registry.
Sampling strategy	As this study was an observatory study from cohort registry that collects data sample size was not calculated. But to ensure normality in the analysis at least 30 participants were thought to be required for each group.
Data collection	Data collection was done by pen and paper and computer as appropriate. As it is an observational study, the researcher was not blind to experimental condition as it was not required in this study.
Timing	Data starts at July 2015 and ends at November 2022.
Data exclusions	No data was excluded from the analyses.
Non-participation	No participants were dropped out during the longitudinal analysis.
Randomization	Participants were not allocated into experimental groups. Appropriate covariate was included in the statistical models to control its influence.

Reporting for specific materials, systems and methods

We require information from authors about some types of materials, experimental systems and methods used in many studies. Here, indicate whether each material, system or method listed is relevant to your study. If you are not sure if a list item applies to your research, read the appropriate section before selecting a response.

Materials & experimental systems

n/a	Involved in the study
☒	☐ Antibodies
☒	☐ Eukaryotic cell lines
☒	☐ Palaeontology and archaeology
☒	☐ Animals and other organisms
☐	☒ Clinical data
☒	☐ Dual use research of concern
☒	☐ Plants

Methods

n/a	Involved in the study
☒	☐ ChIP-seq
☒	☐ Flow cytometry
☒	☐ MRI-based neuroimaging

Clinical data

Policy information about clinical studies

All manuscripts should comply with the ICMJE guidelines for publication of clinical research and a completed CONSORT checklist must be included with all submissions.

Clinical trial registration: *Provide the trial registration number from ClinicalTrials.gov or an equivalent agency.*

Study protocol: Study protocol is not available as this study is founded upon a PD registry cohort.

Data collection: Ppatients presenting between July 2015 and November 2022 were enrolled in this cohort. Data was collected from enrolled patients who visited the St.Mary's Hospital.

Outcomes: The outcome was how ET, PDconv and PD differed in SUVRs of dopamine neuroimaging and how the PD subtypes changed with progression. The subtypes of PD was defined by the presence of antedated ET. In cases of group comparisons, descriptive statistics and independent t-test or Welch's t-test, analysis of variance (ANOVA) or Kruskal-Wallis test were performed for continuous variables. Categorical variables were examined by Fisher's exact test. Mantel-Haenszel test was used to detect any trend between nominal and ordinal scales. Analysis of covariance (ANCOVA) was applied to assess the between-group differences of subregional SUVRs with age at diagnosis as a covariate. Polynomial contrasts were executed to investigate any trends across disease groups: ET vs. PDconv vs. PD. Linear mixed model by restricted maximum likelihood estimation was performed to investigate the pattern of cardiac denervation progression. Intercept was set as random effect to reflect individual variance. Multiple comparisons were adjusted by Tukey or Dwass-Steel-Critchlow-Fligner methods for ANOVA or Kruskal-Wallis test, respectively. In region-based analyses, the false discovery rate (FDR) method was applied for the multiple ANCOVA tests. Scheffe method was utilized when adjusting for the post hoc pairwise comparisons between the disease groups. Statistical significance was defined as a two-tailed p-value < 0.05.
